# The check-up: in-person, computerized, and telephone adaptations of motivational enhancement treatment to elicit voluntary participation by the contemplator

**DOI:** 10.1186/1747-597X-2-2

**Published:** 2007-01-08

**Authors:** Denise D Walker, Roger A Roffman, Joseph F Picciano, Robert S Stephens

**Affiliations:** 1 University of Washington, School of Social Work, Innovative Programs Research Group, Seattle, Washington, USA; 2 Department of Psychology, Virginia Polytechnic Institute, Blacksburg, Virginia, USA

## Abstract

Countless barriers come between people who are struggling with substance abuse and those charged with providing substance abuse treatment. The check-up, a form of motivational enhancement therapy, is a harm reduction intervention that offers a manner of supporting individuals by lowering specific barriers to reaching those who are untreated. The check-up was originally developed to reach problem drinkers who were neither seeking treatment nor self-initiating change. The intervention, marketed as an opportunity to take stock of one's experiences, involves an assessment and personalized feedback delivered with a counseling style termed motivational interviewing. Check-ups can be offered in care settings to individuals who, as a result of screening, manifest risk factors for specific disorders such as alcoholism. They can also be free-standing and publicized widely to the general public. This paper will discuss illustrations of in-person, computerized, in-school, and telephone applications of the free-standing type of check-up with reference to alcohol consumers, adult and adolescent marijuana smokers, and gay/bisexual males at risk for sexual transmission of HIV. The paper's major focus is to highlight how unique features of each application have the potential of reducing barriers to reaching specific at-risk populations. Also considered are key policy issues such as how check-up services can be funded, which venues are appropriate for the delivery of check-up interventions, pertinent competency criteria in evaluating staff who deliver this intervention, how marketing can be designed to reach contemplators in untreated at-risk populations, and how a check-up's success ought to be defined.

## Background

A substantial number of individuals with addictive disorders do not enter treatment. In the U.S. as an example, the 2005 National Survey on Drug Use and Health indicated that while 23.2 million individuals (age 12 or older) needed treatment for an illicit drug or alcohol use problem, only 10.0 percent received treatment at a specialty facility. Remaining untreated were 20.9 million individuals [1]. Most who are experiencing consequences in the early stages of problem development do not consider approaching treatment. Formal treatment is often a last resort *after *the problem has become quite severe and more difficult to treat than if the patient had presented earlier. Non-participation in treatment may involve several factors: lack of motivation or ambivalence, obstacles to access, stigma, or cultural barriers. While momentous life events are sufficient to prompt increased readiness to change in some individuals, much interest exists for developing and testing interventions to accomplish this purpose.

In clinical practice, few, if any, treatment approaches center on attracting and motivating those in the early stages of change. Treatment facilities are by nature a poor match for individuals who either do not currently identify their behavior as a problem (precontemplation) or are just beginning to consider the pros and cons of their behavior but have not decided to change (contemplation). A common sentiment among treatment providers is that patients "need to be ready to change." Consequently, the limited care options available to those with substance use disorders who are also at early levels of readiness for change leave a large percentage of individuals unserved.

One response to this gap involves efforts in primary care settings to identify early those with substance use disorders. Increasingly, health care settings have implemented screening of patients for alcohol, tobacco, and other drug problems. Much of the impetus for this has come from studies that point to the effectiveness of early intervention [2-4].

Another approach involves strategies to assist family members or friends in motivating reluctant substance users to enter treatment [5]. These interventions vary in theoretical orientation and may lead to disparate recommendations made to "concerned others" about how to interact with a loved one who is abusing substances. The spectrum of recommendations varies from "detaching" from the loved one [6] to coordinating a group of family, friends and co-workers or employers to confront the user with the ways in which the substance use has affected them [7]. Operant learning principles as found in Community Reinforcement and Family Training [8,9] and unilateral family therapy have informed the development of other approaches [10-12].

In contrast to the variety of approaches available for concerned others of substance abusers to increase treatment engagement, few intervention options exist for motivating *individuals *who are contemplating the effects of risky behavior on their lives but are neither self-initiating change nor seeking treatment. A variant of motivational enhancement therapy (MET) called a "check-up" shows promise of serving this purpose. Generally involving 1 to 4 sessions, MET commonly includes an assessment, feedback, and an exploration of the client's attitudes favoring and opposing change. Central to MET is a counseling style called motivational interviewing that is client-centered, intended to support individuals in resolving ambivalence, and supports the client in seeing the discrepancy between his/her current behaviors and his/her larger goals and values. Motivational interviewing is designed to enhance the client's commitment to change [13].

As reported thus far in the literature, MET can be of three types, the first two of which are designed for those seeking or being referred to treatment. The first, a brief motivational enhancement treatment, may be sufficient in promoting change and is exemplified by the protocol for a four session therapeutic intervention evaluated in Project MATCH [14]. The second, an integrated motivational enhancement therapy that commonly involves multiple clinical components, e.g. MET, cognitive-behavioral skills training, and case management, is exemplified by the protocol for a nine-session therapeutic intervention tested in the Marijuana Treatment Project [15]. The third, a brief motivational enhancement catalyst (i.e., a check-up), is tailored for the non-treatment seeker, with the intention of promoting a "taking stock" experience designed to increase motivation for change. The motivational enhancement catalyst variant of MET either can be offered to those who first have been screened and found to present selected risk factors (e.g., patients seen in a primary care setting and indicating risk for alcohol disorders) or can be free-standing, with a recruitment process that widely publicizes the service and invites those who are interested to contact the program for more information.

This paper will briefly describe several examples of the free-standing check-up, with an emphasis on highlighting the unique ways in which each variant is intended to increase access for select at-risk populations. This model has been adapted for various target behaviors (problem drinking, marijuana abuse, AIDS-risk sexual behavior), populations (adults, adolescents, men who have sex with men), and modes of delivery (in-person, computerized, in-school, via the telephone). Because this is a developing field of intervention research, this paper will focus primarily on describing the process for engaging at-risk and non-treatment-seeking individuals in free-standing motivational enhancement catalyst interventions. Brief attention will be given to the outcomes from these trials. The ways in which each application seeks to reduce barriers to reaching contemplators will be highlighted and clinical issues and challenges will be discussed.

Whether of the free-standing variety or as a service offered to screened clientele, check-ups share five common elements. First, to enhance the perceived value of the experience by the client, the prospective participant is helped to understand the nature of the check-up as something other than treatment, that may be helpful to the individual who is questioning his/her current behavior or has concerns, and that he/she has the choice to accept or reject feedback offered during participation. Second, each check-up involves an assessment that captures information about behavioral patterns, positive and negative consequences perceived by the client, and attitudes regarding changing or not changing the behavior. Third, check-ups offer personalized feedback to the participant. Commonly, personalized feedback reports (PFRs) are created from the client's assessment responses and include: normative comparison data (e.g., how the individual's drinking frequency compares with the average frequency of drinking by the general population), graphics that are used to enhance self-appraisal of the behavior, risk-related indices, and the client's anticipated positive and negative consequences from changing. Fourth, a style of interviewing that facilitates a candid taking stock of one's behavior (i.e., motivational interviewing) is essential to this type of experience. Finally, motivational enhancement catalyst interventions are informed by harm reduction principles[16] and the stages of change model [17], both of which support tailoring interventions to meet individuals "where they are" in order to reduce stigma associated with help-seeking and to encourage low-threshold access to services as a means of supporting steps (however big or small) toward change.

As will be discussed in further detail below, the free-standing check-up relies on marketing to elicit self-referrals by members of the target population. Advertisements for a free-standing check-up often emphasize that the intervention is not treatment, but a pressure-free chance for those who have concerns to "take stock" of their experiences with the support of a professional. It is made clear that the participant is responsible for deciding what to do, if anything, with the information he/she receives.

### Five variants of free-standing check-up interventions

#### Reaching the adult problem drinker

*The in-person "Drinker's Check-Up." *Several issues may contribute to a lack of treatment initiation for those struggling with alcohol problems. First, the label of "alcoholic" conjures up images of a drinker at the extreme end of the spectrum whose life is centered on obtaining and using alcohol to the severe detriment of health, employment, housing, and relationships. Many individuals who are experiencing alcohol-related adverse consequences do not view these images as relevant to their circumstances, fear the consequences of being labeled, and question whether a commitment to being abstinent is necessary. Anticipating that seeking help will result in being pressured to both accept the alcoholic label and completely abstain may dissuade individuals who are experiencing milder problems related to alcohol from crossing the threshold of treatment programs.

As described by Miller, Sovereign, and Krege [18], the Drinker's Check-Up (DCU) was developed to reach problem drinkers who were not interested in formal treatment but who had some concerns about their drinking. It provided a voluntary opportunity for drinkers to assess how their alcohol use might be influencing their life. In an initial trial with 42 participants, the DCU was promoted as a free assessment and feedback service for drinkers who wanted to find out whether alcohol was harming them. Publicity emphasized the program was not intended for "alcoholics." Its de-emphasis on "alcoholism" was meant to overcome issues of labeling and associated stigma that might dissuade participation by contemplators.

Participants first completed an assessment battery that included a structured interview, questionnaires, and a brief neuropsychological assessment. The second session was focused on giving feedback which included: a comparison of the client's alcohol consumption per week with that of the average American drinker, the peak blood alcohol concentration of the client during a typical week and during heavy periods, the extent of family risk for alcohol problems, and the severity of problems associated with the client's alcohol use related to research norms and cut-points.

The DCU offered the first conceptualization of the check-up modality [18]. The researchers reported that most of those who voluntarily participated were similar to clients already in treatment on measures of alcohol use and related problems. Participants, on average, reported having consumed 44 standard drinks per week, a relatively high consumption level by U.S. standards. However, most did not consider themselves to have a drinking problem. The majority cited personal reasons for becoming involved with the DCU, with only one individual reporting participating because of outside pressures (i.e., concern for a job, pressure from an employer or the courts, or a crisis). Significant reductions (27%) in alcohol consumption and peak blood alcohol concentration (29%) were found for participants at the six week follow-up. At 18 months, consumption had decreased by 29% and peak BAC had reduced by 37% from baseline, respectively. The DCU attracted voluntary participation from heavy drinkers not considering treatment or self-identifying as problem drinkers. Further, the DCU intervention led to self-initiated drinking reductions in a substantial subset of the participants. These results were replicated in a second trial [19].

#### Reaching the adult problem drinker

*The computer-based "Drinker's Check-Up." *Advances in technology have sparked interest in applying technical innovations to clinical practice. Computer-based assessment and screening tools have been developed for an array of problem behaviors and have demonstrated reliability and validity [20]. Computerized treatment interventions also offer promise for various psychological problems including the treatment of agoraphobia, panic disorder, mild depression and reducing behavioral risk for diabetes [see [21] for review].

Kobak et al. [22] evaluated the validity and clinical utility of a telephone-assisted computer-administered psychiatric disorder assessment protocol with 200 primary care outpatients. Computer-based telephone screening using interactive voice response technology was twice as likely to detect alcohol abuse than screening utilizing the SCID conducted by physicians over the telephone (15.0% vs 7.5%). Computerized interventions have been developed for smoking cessation [23] and alcohol moderation [24]. Screening and brief feedback programs have also documented effectiveness for use with alcohol and other drugs [25,26].

Squires and Hester [27] reasoned that a computerized version of the DCU (CDCU) may be an ideal vehicle to offer the DCU to the general public outside of a clinic or other professional setting. Alternatively, a CDCU may aid clinicians in mental health settings who encounter clients with alcohol disorders but do not have expertise in substance abuse brief interventions.

An effectiveness trial of the CDCU has recently been completed [28]. Newspaper advertisements recruited the majority of participants. In addition to the common marketing messages of the DCU, CDCU advertisements provided a foundation for self-efficacy to be built: "Are you concerned about your drinking habits? Did you know that 75% of people who change their drinking do it on their own?"

In contrast to the in-person DCU, the CDCU consisted of one assessment and feedback session conducted entirely via the computer. The intervention took between one and two hours to complete. Three elements made up the program: assessment, feedback, and decision-making modules. The assessment module first administered the Alcohol Use Disorders Identification Test or AUDIT [29] for screening purposes. Individuals who scored eight or higher (at risk or higher) were invited to proceed. Participants registered by providing their first name, a password, gender, weight, and height. Gender, height and weight were required to calculate peak blood alcohol concentrations in the feedback module. Participants then were directed to the Decisional Balance exercise which compared "the good things and the not so good things" about their drinking [30]. Lists of positive and negative aspects about drinking were provided for participants to choose from in addition to the option of typing in their own responses. This exercise is a basic motivational interviewing technique to give voice to a client's ambivalence. Participants' submitted responses were saved and reviewed later in the Decision Making module. Further instruments were then administered.

Following completion of the assessment module, personalized feedback was provided. Automatic links were developed to respond to various reactions participants might experience. Pilot testing with a focus group helped ensure that the links were derived in a motivational interviewing style. The Decision Making module began with a Readiness Ruler and individualized options based on participants' responses. For example, if a participant reported "Not at all Ready" to change, the computer offered the options of printing the PFR or viewing the "Alcohol and You" pamphlet before exiting. If a participant indicated "Really Ready to Change," the program proceeded directly to developing a change plan.

In this effectiveness trial, sixty-one participants were recruited and randomized to receive the CDCU immediately or after a 4-week delay. Immediate participants were assessed at baseline, 4-week, 8-week and 12 month follow-ups. A unique feature of this study included the Delayed group not being assessed until the 4-week follow-up so as not to produce reactive effects to the assessment. The Immediate condition reduced their drinking significantly more relative to the Delayed condition at the 4-week follow-up. After the Delayed group received the CDCU, drinking rates were similar between the groups at the 8-week and 12-month follow-ups. Both groups reduced their drinking by approximately 50% as compared with baseline, and these gains were sustained at the 1-year follow-up. This study supports the promise of utilizing a CDCU for alcohol problems.

#### Reaching the adult marijuana smoker

*The in-person "Marijuana Check-Up." *For those who smoke marijuana and have concerns about adverse consequences, ambivalence about change may stem from contradictory messages available from government programs, peer groups and/or marijuana advocacy groups. On the one side are exaggerated and sometimes false representations of the dangers of marijuana use. On the other side, equally inaccurate projections portray marijuana as a benign substance with no adverse side effects or consequences.

The Marijuana Check-Up (MCU) was developed to attract adults who use marijuana, experience negative consequences from their use, and are not seeking treatment or self-initiating change. It sought to provide an opportunity for individuals to reflect on how their marijuana use was affecting their lives by providing individualized feedback and scientifically based educational information about the drug.

Similar to the DCU, the MCU was advertised as a chance to receive objective feedback about marijuana use. Emphasizing the provision of non-biased information was especially critical given marijuana's history in American culture. Marketing efforts were intended to elicit interest from a wide variety of users.

A controlled trial was conducted with 188 participants to evaluate the efficacy of the MCU [31]. Participants were randomly assigned to one of three groups: MET, Multimedia Feedback (MMF), or delayed treatment (DF). In the MET condition, the personalized feedback report consisted of five sections: Your Marijuana Use, Risk Factors, Consequences of Use, Marijuana Problems, and Confidence in Avoiding Use. The MMF condition was an educational intervention that reviewed research findings on the effects of marijuana presented via computerized slides and a video documentary. DF participants waited seven weeks and then received their choice of the MET or MMF interventions.

Based on a stage of change algorithm the majority of individuals attracted by this study were in the precontemplation (39%) or contemplation (30%), stages of change. On average, participants smoked marijuana on nearly six days per week during the past 90 days and were high six hours on a typical day. When administered the Psychoactive Substance Use Disorders section of the Structured Clinical Interview for DSM-IV [32], they endorsed an average of 3.45 DSM-IV criteria for marijuana dependence. Sixty-four percent met DSM-IV criteria for cannabis dependence, and an additional 29% for cannabis abuse. In terms of their frequency and volume of marijuana use, the sample resembled treatment-seeking participants in another trial. However, they reported fewer problems related to their marijuana use and met fewer dependence criteria [31].

The MET condition reduced their days of marijuana use, periods of use per day, and dependence symptoms relative to the MMF and DF conditions at the 7-week follow-up [33]. MET participants reported fewer dependence symptoms at both the 6 and 12-month follow-ups compared to MMF participants. The MET participants sustained their marijuana use reductions by the 12-month follow-up and continued to use less than either of the control groups. The MCU was successful at attracting ambivalent heavy marijuana users and demonstrated promise as a means for reducing use and dependence symptoms. Although reductions in marijuana use were relatively small, these outcomes suggest that future research is warranted to explore ways to heighten these effects.

#### Reaching the adolescent marijuana smoker

*The in-school "Teen Marijuana Check-Up." *Adolescents pose both similar and unique challenges for engaging those who are demonstrating high-risk behavior. Self-referral is rare among adolescents; rather, they are referred by family, the juvenile justice system, or the schools. When adolescents do enter treatment, few (20%) believe their use is problematic [34]. Adolescents often lack the resources needed to self-refer to treatment (e.g., insurance, finances, transportation). Further, teens whose parents are unaware of their use may be deterred from participating in substance abuse interventions if parental consent is required.

The Teen Marijuana Check-Up (TMCU) was adapted to overcome the challenges identified above. The intervention was intended to elicit voluntary participation from marijuana-using teens. Providing the intervention in school during the regular school day, obtaining a waiver of the requirement to seek parental consent, and assuring confidentiality were some of the ways barriers were reduced for teens to participate in the TMCU.

The delivery of the TMCU in high schools required a distinct marketing strategy. Research staff visited regularly scheduled classes and offered a guest talk that reviewed the effects of marijuana. During these presentations, teens were prompted to engage in a discussion focused on myths and facts about marijuana, the TMCU was described, and the opportunity was given to indicate interest in the study by writing their names on an otherwise anonymous evaluation form at the end of the class period. Staff presented information about marijuana and its effects in an unbiased manner and acknowledged there may be benefits from marijuana use. In this way, the students were exposed first-hand to the nonjudgmental and objective manner in which the TMCU intervention was delivered. Additionally, advertisements were placed throughout campus similar to those used for the MCU. Considerable effort was also directed toward educating school personnel about the project to promote referrals. School staff members agreed that the identities of students who participated would remain confidential among project staff.

The TMCU intervention aimed to facilitate the adolescent's candid exploration of his/her marijuana experiences including costs and benefits, comparisons of one's use patterns with those of other teens, and the impact of use on the individual's larger goals and relationships. Additionally, it sought to prompt elaboration and exploration of ambivalent attitudes concerning marijuana use and, when appropriate, to offer support in goal setting and strategies for change.

To reduce response bias and administration time, the TMCU assessment was computerized and self-administered. Personalized feedback reports were printed by the counselor directly from the computerized assessment. In the second session, the teen and counselor reviewed the personalized feedback report. If the student indicated an interest in reducing or quitting marijuana use, a "Strategies that Work" booklet was reviewed that offered tips for making change.

A pilot study and initial efficacy trial have been conducted to develop and preliminarily evaluate the TMCU. The first study explored recruitment methods and the feasibility of attracting non-treatment seeking teens in a single condition pre-test/post-test design [35]. Fifty-four adolescents participated in the two-session (assessment and feedback) TMCU and completed a 3-month follow-up assessment. At baseline, participants reported using marijuana on average 10.7 days of the last 30. At follow-up, significant reductions in marijuana use were reported overall and among heavier users. Reductions in use were reported at 3-months by 54%, and nearly 15% had been abstinent for the past 30 days. Most (98%) reported the TMCU was helpful. Participants also felt their counselor had listened to them (98%) and had been nonjudgmental (91%).

Similar findings were reported in a subsequent controlled trial focused on regular users [36]. This project recruited and randomized 97 adolescents to receive the TMCU immediately or after a three month delay. In an effort to recruit a heavier using sample, the inclusion criteria were modified to include adolescents who had used marijuana on nine or more of the past 30 days prior to baseline, with this frequency level serving as a proxy for greater than weekend-only use. Participants' marijuana use, related consequences, and expectancies were assessed at baseline and at a 3-month follow-up. Responding to a 5-part algorithm with one statement that corresponded to each of five stages of change, two-thirds of the participants categorized themselves as in the precontemplation or contemplation stages of change regarding marijuana use. Approximately a third (34%) of the participants reported having had prior treatment for drug or alcohol use. At baseline, participants reported using marijuana an average 38 days of the last 60. Analyses revealed both groups significantly reduced their use of marijuana at the 3-month follow-up. Post-hoc analyses suggest that the Immediate condition was associated with greater reductions in use for participants in early stages of change. Overall, the TMCU was successful in recruiting marijuana-using adolescents to voluntarily participate. Preliminary evidence indicates the TMCU aids in decreasing marijuana use among adolescents. A currently underway efficacy trial is seeking to evaluate the TMCU as a means of engaging teens in treatment aimed at achieving abstinence.

#### Reaching the gay or bisexual male at risk of HIV transmission

*The telephone-delivered "Sex Check-Up." *Within HIV and STD prevention programming among men who have sex with men (MSM), several subtle and complex barriers exist for accessing services. Traditional risk-reduction counseling may not reach individuals at greatest risk [37]. Of those reached, as many as half of the individuals who are interested and eligible to participate in HIV interventions do not complete treatment, and 20% who enroll never present for the intervention [38-41]. Evidence indicates concern over HIV and AIDS is declining among those who perceive undetectable viral loads as non-infectious and among individuals who believe that HIV is less dangerous given recent advances in pharmacological therapy [42]. Minority subgroups within the MSM population such as men of color, HIV-positive individuals, men who are older, adolescents and bisexuals may not feel that staff of prevention programs are adequately prepared to understand their distinct situations. Closeted individuals may fear participation in risk-reduction programs could compromise their anonymity and privacy. Others who are active in the gay community in various helping or activist positions and who are struggling to be sexually safe in their own lives may avoid seeking support for fear that it may compromise their position in the community.

The Sex Check-Up or SCU [43], another free-standing motivational catalyst adaptation, was developed with many of the above barriers in mind. When a more intensive telephone-delivered HIV-prevention counseling service was previously offered by this research group, only a third of individuals who contacted the project and were screened and found eligible actually enrolled [40]. The investigators posited that many of those who approached the project but then discontinued involvement might have been struggling with ambivalence.

Seeking to draw involvement from MSM who were concerned about their high-risk sexual behavior was therefore the goal of the Sex Check-Up. To lower access barriers, the SCU was delivered over the telephone in a brief treatment. A telephone intervention has the unique potential to reach those with geographic, mobility or transportation concerns and substantially reduce trepidation related to privacy. Anonymous enrollment was also made available. Participants choosing this option were asked to use a pseudonym when renting a post office box (for which they were reimbursed) to provide a means for transmitting project materials and incentive payments for follow-up interviews to them [44].

Positing that many MSM would have conflicting feelings about safer sex and that some may eschew prevention programs that imply a demand for behavior change, the SCU publicity described the intervention as an opportunity to talk with a nonjudgmental and supportive counselor about one's ambivalent feelings regarding sexual safety. Ambivalence was directly portrayed in advertisements by such quotes as "I really have mixed feelings about always needing to be safe" and "I know what I do is risky." Attention was drawn to how the SCU differed from other prevention services offered in the community: "It's not HIV education. It's not a condom workshop."

Delivery of the SCU over the phone required a few adaptations from conventional in-person counseling. Body language and facial expressions could not be seen to gauge client comfort. Verbal language or the lack thereof was relied upon to inform the clinician of the client's readiness to progress through topics and sections of the PFR.

Results from a randomized controlled trial comparing the SCU to a delayed counseling control group indicated men at risk for HIV infection could be attracted to participate in the intervention [45]. The majority of men (55%) were in the precontempation or contemplation stages of change at the baseline assessment. Compared to the delayed control condition, the SCU led to an increase in motivation to change risky sexual practices and had a differential impact on reducing unprotected anal intercourse among men of color.

### Clinical challenges

#### Publicity and recruitment – how do you get contemplators to participate?

One of the most striking aspects in reviewing applications of the free-standing motivational catalyst check-up is that well constructed publicity messages prompt high-risk individuals to respond to and enroll in this type of intervention. Among the various applications, over 500 participants volunteered to participate in a check-up experience. The majority of these participants heard about the check-up through published advertisements or presentations. What are key aspects of effective advertisements? Publicity that normalizes the help-seeking process appears to be productive. Emphasizing that the check-up is "not treatment," but rather "an opportunity for you to find out more about how your behavior is affecting your life" is intended to convey that individuals will not be asked to commit to either change or to a lengthy treatment program. The check-up is characterized as an opportunity to "sample" what talking with someone about a behavior is like without pressure to continue. Advertisements are label-free. Individuals do not have to admit they are a problem drinker or marijuana dependent to explore if change is right for them. Similarly, a de-emphasis on treatment reinforces this aspect. Confidentiality is respected.

#### Training and supervision of staff

There is a delicate balance to be struck in providing a "pressure free" intervention, and fostering motivation to change. Within check-up interventions, staff seek to provide an environment where individuals who are ambivalent about change can objectively examine their behavioral choices, with the clinician carefully listening for statements indicating motivation to change. Competence in motivational interviewing skills rather than client behavior change needs to be the measure of counselor competence used in supervision. This can be accomplished by utilizing tape coding systems such as the Motivational Interviewing Skill Code or MISC [46] or adaptations of the MISC such as the Motivational Interviewing Treatment Integrity code or MITI [47]. These systems offer objective behavioral feedback to counselors regarding motivational interviewing skills and can assist in reinforcing counselors for maintaining intervention fidelity rather than eliciting client commitment to change. If executed properly, the two should be closely linked. However, equating therapist competence with client commitment to change could jeopardize the spirit of the delivery of the check-up intervention.

It can be difficult to train staff in the nuances of motivation and its manifestations in clients. Accurately recognizing when a client is ready to move forward toward change is a higher level skill. Making this a specific training goal is a necessary aspect of a training program. Reviewing client transcripts and tapes for self-motivational statements can be helpful toward this end. More needs to be learned about the optimal timetable and curriculum for training of clinicians who deliver check-up interventions.

#### PFR – mail delivery or in-person

*Does it create reactance? *Between the various applications of the check-up described, one way in which the SCU differed was that the SCU mailed the PFR to the participant prior to the conversation with the counselor. This was done out of necessity because the SCU was a phone-delivered intervention. This raises the issue of whether or not these modes of delivery are equivalent. Does receiving one's PFR via the mail with the opportunity to review it prior to the meeting affect the content of the meeting, attendance, or other aspects of the experience? Mailed feedback in the absence of an interaction with a counselor has been shown in MET interventions with college student drinkers to be more effective than in-person feedback delivered in a group [48,49]. In contrast, one study with domestic violence shelter residents found written feedback on substance use risk factors delivered to shelter mailboxes produced negative reactions and outcomes in participants compared to in-person feedback [50]. These findings suggest that the mode of delivery of feedback may be an important factor and should be explored further. A related question is determining the relative impact of PFRs when delivered by counselors who have been or have not been trained in motivational interviewing skills.

#### What is a successful outcome?

Success is more broadly defined in check-ups than is the case with other interventions. Any movement toward a healthier lifestyle is considered success. This includes if a client seeks formal treatment, self-help support, or changes on their own. Change also wears a variety of hats including abstinence, reduction in use, or reduction in problems associated with use. Squires and Hester [27] report three case examples of the CDCU leading to seeking formal and informal support services. Most of the studies reviewed above report significant reductions in substance use and risky behavior without additional help beyond participating in the check-up. Both types of outcomes are considered positive.

#### Funding – who will pay for check-up services?

Many of the check-up services described have only been provided as part of research programs and thus were offered free of charge. This has likely added to the appeal for participants to "try out" this type of service. Studies in this area are needed to determine if clients will pay for a check-up. The potential for the check-up qualifying for third-party reimbursement is also important to determine. Although check-up interventions appear to have the potential of being cost-effective due to their brevity, considering ways to reduce such costs even further may help with the dissemination of these interventions. The in-person and over the phone check-up interventions require potentially costly personnel. Assessments alone have lasted from one to four hours. Computerized assessment as created and described in the TMCU offers a less costly means of delivering the check-up, eliminating in-person assessor time. Similarly, fully computerized versions of check-ups have the potential for reducing the costs even further. When offered in care settings, check-ups can offer an ideal complement to other services in primary health care, mental health clinics, and probation and parole settings. Research is needed to determine if computerized check-ups are feasible during the time patients wait for other types of health care or social services appointments. Web-based versions may also provide a cost-cutting alternative if shown to be efficacious.

#### Ethics

There may be ethical considerations for treatment agencies offering check-up services in addition to regular treatment services. On the one hand, offering check-up programs addresses the needs of those individuals who are earlier in their stage of change. Housing both check-up and treatment services in one agency may be of benefit to a client who feels connected to the agency and would like to proceed to a higher level of care based on that experience. This might decrease barriers to treatment entry by reducing the discomfort that may accompany having to research other agencies and services. On the other hand, the treatment agency may directly benefit from increased motivation to change for clients who choose to enter formal treatment. Such agencies may be viewed with suspicion by potential clients who believe the ulterior motive for the facility to offer check-up services is to "drum up" business. In such contexts, care should be taken to provide check-up clients with a comprehensive menu of treatment options and self-help programs in order for the "spirit" of the check-up to be fully realized. A direct statement that acknowledges the potential conflict of interest should be reviewed at the beginning of a conversation about treatment options. These options should be discussed in an objective manner that emphasizes to the client that it is their choice and responsibility to pick a program that best suits their needs.

## Conclusion

Expanding service provision to the millions of individuals who, in the face of substantial risk of harm, do not seek treatment for substance use disorders or other risky behaviors remains a priority challenge. The free-standing check-up model offers a possible blueprint for such an approach. Research has demonstrated the check-up model is a viable approach for attracting high risk groups and the effects on target behaviors are promising but variable. We suspect that the clinical significance of these effects will improve with future iterations of this model.

Additional research will be needed to address questions regarding the effects of feedback components relative to counseling styles, in-person versus computerized formats, the effect of PFRs being mailed in advance as in the SCU study, the extent to which feedback to the client must include new information that the client hadn't known (i.e., cognitive impairment associated with alcohol abuse), the optimal settings in which to embed check-ups, and the optimal training protocol for practitioners who deliver this intervention. However, it already seems clear that check-ups provide opportunities to engage individuals who are in earlier stages of change in secondary prevention and harm reduction.

## Competing interests

The author(s) declare that they have no competing interests.

## Authors' contributions

DDW participated in conceptualization of manuscript, drafted the manuscript, and conducted the literature review. RAR conceptualized the manuscript idea and assisted in drafting the manuscript. JFP reviewed the manuscript and assisted with revisions. RSS assisted with reviewing and editing the manuscript with particular emphasis on intellectual content. All authors read and approved the final manuscript.
